# Understanding compassion for people with dementia in medical and nursing students

**DOI:** 10.1186/s12909-019-1460-y

**Published:** 2019-01-25

**Authors:** Ben Bickford, Stephanie Daley, Gillian Sleater, Molly Hebditch, Sube Banerjee

**Affiliations:** Centre for Dementia Studies, Brighton and Sussex Medical School, Trafford Centre, Univeristy of Sussex, Falmer, East Sussex BN1 9RY UK

**Keywords:** Dementia, Compassion, Empathy, Healthcare students, Medical students, Nursing students

## Abstract

**Background:**

Compassion is an essential component of good quality care. Compassion towards people with dementia in health systems is often suboptimal, which can have negative impacts on clinical outcomes and patient experience. Attitudes are formed early in training and the literature on healthcare student compassion towards those with dementia is limited. This study aimed to understand how undergraduate medical and nursing students understand compassion towards people with dementia and factors influencing the delivery of compassionate care.

**Methods:**

Nine individual in-depth interviews and two focus groups were undertaken with 23 medical and nursing students. A topic guide was developed, and transcripts were analysed using thematic analysis.

**Results:**

The analysis identified three themes which students used to define compassion: (i) connection, (ii) care, and (iii) respect. Three factors were identified as being either facilitators or barriers to delivering compassionate care to people with dementia: (i) patient factors, (ii) student factors, and (iii) connection. Patient factors related to the presence of behaviours which might be challenging to manage. Student factors included student exposure to dementia, as well as student knowledge and skills. Connection focussed on whether there was an awareness and understanding of the person behind the diagnosis.

**Conclusion:**

Undergraduate healthcare students are the future workforce for patients with dementia, and understanding how compassion develops within them is important. We found medical and nursing students had a broad understanding of compassion, and identified factors influence their compassion towards people with dementia. These novel data can be used to shape healthcare education programmes aimed at improving dementia care.

## Key points

- Compassion towards people with dementia is often suboptimal, which can impact negatively on clinical outcomes and care.
- The literature on compassion, and student development of compassion towards people with dementia is limited.
- This study identifies that nursing and medical students define compassion in terms of: connection, care, and respect.
- This study identifies three factors which positively and negative influence compassion towards people with dementia: patient factors, student factors, and connection.
- These findings can inform the content of undergraduate healthcare education and future research.

## Background

Compassion is widely recognised as an essential component of good quality care, but despite it being a commonly used concept in healthcare, the construct of compassion is complex with multiple definitions [1]. Recent research has started to explore these definitions and how specific factors, situations, patient groups, and healthcare providers relate to compassion [1, 2]. Etymologically, the Latin roots of the word ‘compassion’ include the verb ‘patior’ (to suffer) and the affix ‘cum’ (with) [3, 4]. In healthcare, compassion can be defined as ‘a virtuous response that seeks to address the suffering and needs of a person through relational understanding and action’ [5]. It has been suggested that such action ‘can be as quick and as easy as a gentle look or a reassuring touch’ [6]. In a study where compassion in physician-patient dialogues was investigated through recordings, researchers identified three components: recognition, emotional resonance, and movement towards addressing suffering [7]. Healthcare professionals have been found to conceptualise compassion as ‘acting with warmth and empathy, providing individualised patient care and acting in a way you would like others to act towards you’, [8] including ‘perspective taking’, demonstrating empathy, and acting to alleviate suffering [9].

Compassion is rightly expected by patients, professional organisations, and healthcare providers [10–12]. UK professional governing bodies such as the Nursing and Midwifery Council and the General Medical Council stipulate the need for compassion, care, and respect for patients [11, 12] with healthcare providers expected to carry out their roles, not only with expertise and skill, but also with compassion. Conversely, a lack of compassionate care can lead to patient suffering and poor outcomes. This is well illustrated by the UK Francis Report which looked into care at the Mid-Staffordshire National Health Service (NHS) Trust [13]. It identified that high levels of patient suffering, mortality and morbidity were linked to a lack of compassionate care, and concluded that to improve patient outcomes, there was need for more patient-centred and compassionate care [13].

Compassion is of particular importance in the delivery of good quality care to people with dementia. Dementia is a major developing twenty-first century health and care priority. It is estimated there are 50 million people with dementia globally, costing US $1trillion [14]. These numbers and costs are set to increase threefold by 2050. The syndrome is characterised by progressive decline in cognitive function including communication abilities, and increasing activity limitation. Added to these are frequent neuropsychiatric symptoms such as depression, agitation, aggression, and psychosis which may occur at any stage of the illness. Taken together, these clinical characteristics present a particular challenge for health and care services and those who provide them. Gold standard ‘person-centred care’ is underpinned by empathy towards the person with dementia and is reliant upon direct and meaningful engagement, with value being attributed, and the ability to view the world from their perspective [2, 15, 16]. However, the healthcare experiences of many people with dementia in the community and in hospital are often poor [17] with an over-reliance on task-oriented, as opposed to compassionate care [18].

Given the increasing prevalence of dementia and the high multimorbidity associated with the condition, future clinicians, regardless of specialty (other than obstetrics and paediatrics), will be caring for people with dementia [19], and will need to deliver compassionate care. Early clinical experiences (positive or negative) and professional training can shape the career-long attitudes, empathy, and preferences of nursing and medical students to working with particular patient groups [20, 21]. It has been suggested that tailored dementia education could be used as a method to enhance empathy and compassion towards people with dementia before attitudes become fixed [22]. However, there is a lack of empirical evidence on how compassion and empathy towards people with dementia develop in healthcare professionals to inform the content of such educational programmes and many students have little contact with people with dementia [21]. We therefore designed this study to explore how undergraduate medical and nursing students, who had been exposed to contact with people with dementia as part of the Time for Dementia programme, define and understand compassion in their interactions with people with dementia and factors influencing the delivery of compassionate care.

## Method

### Sample and setting

Participants were the nursing students at the University of Surrey in the second year of their 3 year training, and medical students at the Brighton and Sussex Medical School in the third year of their 5 year training. All had participated in the Time for Dementia programme as part of their curricula. The Time for Dementia programme involves students undertaking informal home visits to a family affected by dementia over a 2 year period, along with supporting lectures and workshops [22]. Information about the study was provided to potential participants, and informed written consent was obtained from those taking part in the study. A purposeful sample with an equal number of nursing and medical students were invited to take part in individual qualitative interviews or focus group discussions.

### Procedure

NHS Research Ethics approval was obtained for the study. Interviews were arranged at a time and venue which was suitable for participants. Three researchers (BB, MH and GS) undertook interviews with participants, and programme specific focus groups were held at university sites facilitated by the same researchers. Interviews lasted between 45 and 60 min and the focus groups up to 90 min. A topic guide was developed from a review of the literature, and was used to inform the interviews and focus groups. The topic guides explored the following areas: definitions of compassion; feelings towards people with dementia; preferences for working with people with dementia; and factors enhancing or hindering compassion towards people with dementia. All interviews and focus groups were audio recorded, transcribed verbatim, and checked for accuracy.

### Analysis

Initial analysis included descriptive coding where two researchers (BB and SD) independently coded meaningful segments of text from three transcripts (two interviews and one focus group) to identify possible themes. The two researchers then met to review and discuss coding and agreed on an initial coding framework. The remaining transcripts were coded using constant comparison techniques, which included reviewing coding and data between existing and new transcripts [23]. This allowed identification of new codes, checking consistency in the use of codes, and exploration of the relationships between different codes. The findings from the initial coding were reviewed to identify the most pertinent themes. The two researchers met regularly to identify and discuss emerging themes, and a focussed coding framework was established. Additionally, as one of the researchers (BB) was a medical student, academic supervision was provided by an expereinced non-medical/nursing qualitative researcher. Reflexivity was supported by academic supervision and the use of a fieldwork diary by BB so that he could reflect on the impact of his role (as a medical student) on the research. Additionally, to reduce the risk of bias, BB was not directly involved in the interviews or focus group for medical students. Unfortunately, a similar opportunity for the involvement of a nursing student did not exist. The analysis was supported by the use of Nvivo 11 (QSR International, 2010).

## Results

### Participants

Nine individual interviews and two profession-specific focus groups took place, involving 13 medical students and 10 nursing students respectively. The demographics of participants are shown in Table 1.

**Table 1:** Demographics of participants (*n* = 23)

Characteristics	Median	Range
Age	23	20–47
		Number (%)
Gender	Male	7 (30)
	Female	16 (70)
Participant type	Medical student	13 (57)
	General (adult) nurse	8 (35)
	Mental health nurse	2 (9)

### Qualitative findings

The analysis included student definition of compassion and factors they identified as influencing compassion towards people with dementia. There was consistency in themes across the medical and nursing students.

### Defining compassion

The analysis identified three broad themes which students used to define compassion: (i) connection, (ii) care, and (iii) respect. While the three themes are reported separately, they were frequently discussed together.

**Connection** involved being able to understand and recognise the situation which the person with dementia was in, as well as demonstrating empathy by being able to put oneself in the patient’s place and being able to communicate an understanding of their struggle.

*“Realising how frustrating it must be for them* (the person with dementia) *… not being able to remember a certain thing and it must be very, very frustrating.”* (Interview nine, nursing student).

In the **Caring** theme the participants perceived that caring involved showing kindness, having the motivation to help the person with dementia, and taking actions that responded to suffering by acting to support patients.

*“Compassion is caring about a person but not just because you feel that you need to take care of that person, it’s because, kind of, you want to help. It’s very easy to see for example compassionate care and care without compassion. Care without compassion is more just let’s do it because we have to do it or because we are here to do it or we are paid to do it. It’s* (compassionate care) *more of a desire to help someone.”* (Interview one, nursing student).

**Respect** included recognising the person with dementia’s humanity, maintaining their dignity, and treating them with honesty and patience. It also involved listening, demonstrating a non-judgmental approach, and recognising the person with dementia as separate to, and more than the presenting illness.

*“They* (the person with dementia) *deserve to be treated like a person...it’s very important just to remember that they are still a human and to treat them as appropriate.”* (Medical student focus group, participant eight).

*“Listening, because they still have a voice, even though they have dementia...you know, make them feel like a person still.”* (Nurse focus group, participant five).

### Factors influencing compassion towards patients with dementia

Three key factors were identified when participants were asked about barriers and facilitators influencing compassion towards people with dementia: (i) patient factors, (ii) student factors, and (iii) connection.

#### Patient factors

A recurring theme within the interviews and focus groups was that compassion can be reduced if people with dementia are perceived in terms of their being violent, aggressive, frustrated, or distressed.

*“It’s just come back to me. In my first year I was doing a night shift and I came for early shift, and because this lady became violent and the way the handover was it’s like she just wanted to harm everybody. And yeah, I don’t think that she was treated at that time with compassion.” (Interview seven, nursing student).*

Participants described that when a patient was distressed they or their peers would sometimes choose to avoid the patient or not help them.

*“She* (another medical student) *was walking past and one of the patients shouted at her, for no reason, and she just, from then on, was like I’m out of here, I’m not doing this anymore”. (Interview five, medical student).*

Some participants described feeling indifferent towards patients’ concerns and seeing the only the disease not the person behind the disease. Again, this led to a lack of compassion and lack of action to respond to their distress.

*“I think let’s say a patient* (with dementia) *was shouting, you know, for hours and hours on end, you kind of just tend to tune it out.” (Interview six, nursing student).*

*“You do see that sometimes that people with dementia do like to shout out, I think it’s quite common that they shout for mum…* (and other staff were saying*) “Oh, that’s just them being them, just leave them to it”. So, they know that this is what they do. If we don’t calm them down in five minutes they’re going to be distressed again.” (Medical student focus group, participant eight).*

Finally, participants described difficulty with not being able to cure patients and from this, a feeling of hopelessness and therapeutic nihilism about people with dementia, both of which reduced compassion.

#### Student factors

Participants reported that factors which influenced compassion towards patients with dementia included the amount of experience they had with people with dementia, student knowledge, clinical and communication skills, and the presence of negative emotions.

Increased experience of people with dementia, whether in a personal or professional capacity was seen as facilitating compassion towards those with dementia. Conversely, some participants felt that a lack of experience with dementia care was a barrier to being able to be compassionate towards a person with dementia. Participants identified that gaining experience of dementia care was a facilitator for compassion.

*“I don’t think it’s something that can be taught in a lecture or things like that, you can be aware of the signs and symptoms but unless you actually meet with someone and engage with them you don’t know what it is, what dementia is or what it’s like for someone living with it.” (Interview nine, nursing student).*

Building on familiarity, participants reported that having sufficient knowledge and understanding of the condition and possessing adequate clinical and communication skills enhanced compassion, and conversely that a lack of knowledge might impede compassion.

*“Maybe not having the knowledge of, I don’t know,* (or*) experience. That you don’t recognise what is happening with that person and you might think they may be rude but actually he might be just, you know, he needs a reassurance.” (Interview seven, nursing student).*

Participants reported that their own emotions, particularly the presence of negative emotions could act as a barrier to being compassionate towards people with dementia, even if the cause of their negative emotions was un-related. Participants discussed how feeling hurried, impatient, and overwhelmed with lots of tasks negatively impacted upon their compassion towards people with dementia. Participants also reported that feeling anxious or stressed reduced their compassion for people with dementia, and instead led to frustration towards them.

*“Maybe for them* (people with dementia) *being annoying in a way of, as I said in, if we are talking about looking after patients with dementia in hospital bed settings because you need to do lots of other things that are waiting for you and it can be quite agitating that you can’t really sit down and spend time with the person and then you can kind of lose that compassion towards that person because you are trying to kind of calm them down but it doesn’t really help and bells are ringing. And then just because of the busyness for example in an area it, compassion can be lost in a way.”* (Interview one, nursing student).

#### Connection

Connection was perceived as both a barrier and facilitator to compassion. Knowing and understanding the person behind the illness, and having knowledge about their life, their family, and their preferences was seen as enhancing compassion, whereas a lack of connection diminished it.

*“I really like those hospital passports that some of the wards do, here, and I guess other places. So, you’ve got a bit of blurb about what the person in front of you,* (their) * dislikes and likes, because you might not be able to get that out, you know, extract that from the conversation straight away, so at least you then have something to start a conversation about, that’s not medical.” (Medical student focus group, participant eight).*

*“I don’t think I’d necessarily call it not being compassionate but it’s the fact that when you’re potentially on a ward round, something like that, you’ve got your 30 patients that you have to see and you’ve just grabbed their drugs chart, it looks fine, you move on, you haven’t really built that connection and I think that is where some of us might feel that we need to work on...so, potentially you never really get to know what that patient needs from a non-medical side.” (Medical student focus group, participant six).*

Understanding the impact of the condition on the person’s life and their family facilitated connection between the participants and their patients. Appreciating the personal impact of illness enhanced compassion towards people with dementia.

*“To see someone crying their eyes out because they can’t cope, adds an aspect that no textbook’s going to give you and if that doesn’t do something to you to make you feel compassion, you are in the wrong career, definitely wrong career.” (Interview three, medical student).*

## Discussion

This study is the first to explore in-depth student compassion in the context of caring for people with dementia. The three student definitions of compassion (connection, care, and respect) and the three identified barriers/facilitators to compassion towards people with dementia (connection, patient factors, and student factors) are of potential help in assessing potential impacts of education and training on dementia care and in designing such programmes.

As discussed above, compassion is a complex concept. Our findings revealed that healthcare students view compassion as connecting with a patient and understanding their situation, and that caring and kindness are key components of compassion, all of which are consistent with the wider literature on conditions other than dementia [8, 24]. Our finding that connection and student and patient factors influence compassion towards people with dementia is consistent with The Transactional Model of Physician Compassion which depicts facilitators and barriers to compassion as a function of clinician, patient and system factors [24].

In terms of student factors, we found that level of exposure through personal or professional experience of dementia can either facilitate or hinder compassion. One study exploring whether compassion could be taught to medical students found that personal experience could be a key contributor to compassion [25]. Nursing research has suggested that previous experience of dementia can be helpful when caring for people affected by advanced dementia [26]. Personal experience of the illness, has also been found to help develop compassion [27]. This suggests that there is value in programmed time spent with people with dementia and their families as part of undergraduate healthcare education.

Having accurate knowledge about dementia was seen as facilitating compassion towards patients with dementia. Consistent with this, it has been found that a lack of knowledge and understanding about dementia can negatively impact care of dementia patients [19, 26, 28]. In addition to having knowledge about dementia, clinical and communication skills were perceived to facilitate compassion. This is consistent with the wider literature on the delivery of care to people with dementia, where having these skills has been seen as enhancing compassionate care [29], whereas lack of skill in these domains is recognised as adversely affecting care [6, 26, 30, 31].

Our study found that negative student emotions, and situational stress impeded compassion. This finding is similar to studies which found that a negative emotional state could reduce compassion [32] and empathy towards a person with dementia [33]. The busy nature of clinical work and lack of time has been shown to impact negatively on clinicians’ emotional state therefore reducing compassion and quality of care [30, 32, 33].

Our findings did not identify reflection as either a barrier or facilitator to empathy towards people with dementia. This is not in keeping with wider literature [25, 34], for example, one study looking at student nurses suggested that self-reflective opportunities were an influencing factor of compassion [34]. Further, it has been suggested that enhanced compassion involves experiential and reflective learning from life and clinical experiences [25, 27]. We also found no reference to role modelling which has been identified as an important method of enhancing compassion [25]. It may be that the integral reflection that is part of the curricula at the medical and the nursing school studied means that there was little variation in reflection so it was seen as normal practice. In terms of role modelling it may be that the powerful educative role that people with dementia and family carers take up in the Time for Dementia programme so that compassion and understanding are forefronted, again resulting in saturation and a consequent lack of variation in our sample.

This study suggests that patient behaviour, when not understood as part of the illness, was a barrier to compassion. Staff may be reluctant to engage with aggressive patients [33, 35] and when people with dementia are aggressive or distressed this may cause negative impact on the care delivered [36]. These types of attitudes towards the behaviours of dementia patients are not uncommon [36–39]. Obtaining personal information on the person with dementia and clinical knowledge were seen as effective in addressing these negative perceptions and promoting compassion, enabling students to recognise the person behind the disease. This demonstrates the value of ensuring that students understand that the behaviour of people with dementia is not in their control but is part of the disease or a demonstration of unmet need [38].

However, the findings from this study also show how, despite an understanding of compassion and increased dementia education, people with dementia may continue to be perceived negatively [40–42], even in healthcare professionals in training, whose views are only beginning to be formed. This fits with Kitwood’s theory of malignant social psychology whereby staff behaviour can undermine personhood and wellbeing in dementia [39]. In terms of addressing this, as noted above, knowing more about the patient and understanding the impact of the illness was a factor influencing compassion positively. This echoes nursing and palliative care research which has shown that seeing patients as unique individuals, is a key component of being compassionate [43, 44, 45]. Within an acute setting, it is recognised that this might be challenging, especially where communication is limited [46], .therefore this strengthens the need to actively engage with family members and use written tools such as personalised information booklets for patients with dementia, which provides information on care preferences, key relationships, in order to enable healthcare providers to connect with the people with dementia under their care.

Concerns have been raised about the limited time in undergraduate healthcare student curricula dedicated to developing skills in and understanding of dementia [47]. Our data suggest that a more nuanced approach education and training in dementia may be required to enhance compassion towards people with dementia [40, 41] Further research is needed into how compassion and empathy develop over time in students at undergraduate level, how this differs between professional groups and how this changes as they move into practice and employment, in order to identify the most effective and timely educational interventions.

There are five main limitations to this study. First, this study was completed in a set of students who had participated in the Time for Dementia programme. This programme will have shaped their beliefs about dementia and compassion and different findings may have been generated from students receiving traditional healthcare education. However we chose this population since we were interested in their beliefs having had contact with people with dementia. In many courses it would have been possible that students would have had no direct experience of dementia. Second the sample included only nursing and medical students, therefore the findings cannot be generalised to other healthcare student groups such as allied health professionals or paramedics. Finally, the majority of the participants were between the ages of 18 to 25, therefore we did not hear perspectives from older students who may have had different perspectives of compassion in dementia care. Fourth, we have not specifically sought to explore the differences between medical and nursing students, and it may be that our results may have been influenced profession specific factors as such as the lack of a nursing researchers, focus of the topic guide and environment, this would need to be considered in future research. Finally, a medical student was part of the research team, but not a nursing student, this may have unintentionally introduced bias into the analysis, although this was partially address by the analysis being led by a researcher with an occupational therapy background (SD).

## Conclusion

This is the first study to investigate directly healthcare students’ perspectives on compassion in dementia care, as such the novel data generated have the potential to inform education and further research. The study has focussed simultaneously on both medical and nursing views and as such takes a bottom up approach to deliver an interdisciplinary perspective which may well be particularly useful in dementia care which requires high levels of multidisciplinary cooperation.

## Abbreviation

NHS: National Health Service
